# Non small-cell lung cancer with metastasis to thigh muscle and mandible: two case reports

**DOI:** 10.1186/1752-1947-7-98

**Published:** 2013-04-08

**Authors:** Francesca Maria Giugliano, Domingo Alberti, Giovanna Guida, Giampaolo De Palma, Luciano Iadanza, Maria Mormile, Fabrizio Cammarota, Agnese Montanino, Franco Fulciniti, Vincenzo Ravo, Paolo Muto

**Affiliations:** 1 Radiation Therapy Department, National Cancer Institute, Pascale Foundation, Naples, Italy; 2 Medical Oncology Unit, Thoraco-Pulmonary Department, National Cancer Institute, Pascale Foundation, Naples, Italy; 3 Pathology Unit, National Cancer Institute, Pascale Foundation, Naples, Italy; 4 Medical Physics Department, National Cancer Institute, Pascale Foundation, Naples, Italy

**Keywords:** Non-small-cell lung cancer, Muscle metastasis, Bone mandible metastasis, Radiation therapy

## Abstract

**Introduction:**

Lung cancer is the leading cause of cancer-related death in Europe and the US. Isolated metastases to skeletal muscle and the mandible are very uncommon.

**Case presentation:**

This report presents two cases. Case 1 concerns a 45-year-old Caucasian woman affected by muscle metastasis of the right thigh from non-small-cell lung cancer. Case 2 concerns a 61-year-old Caucasian man affected by mandible metastasis from non-small-cell lung cancer. Both metastases were detected by diagnostic imaging studies. Both patients were treated with radiation therapy with palliative and antalgic intent.

**Conclusion:**

Radiation therapy was effective and well tolerated in both cases. Both our patients are alive, with follow-up of 18 months and five months, respectively.

## Introduction

Lung cancer is the leading cause of cancer-related death worldwide and the second most common cancer in both men and women. Despite improvements in imaging technologies over the past two decades, the majority of lung cancers are discovered because of the development of distant metastases. Hematogenous spread with multiple organ involvement is frequently reported. Commonly, metastases from lung cancer involve the liver, adrenal glands, bone and brain [1]. Muscle metastases are uncommon [2]. Mandible metastasis from lung cancer is a rare condition that may occur in the late stages of the disease [3]. We describe two cases of non*-*small-cell lung cancer (NSCLC) metastasis to thigh muscle and mandible bone (as first clinical evidence), and discuss treatments and outcomes.

## Case presentation

### Case 1

**Figure 1:**
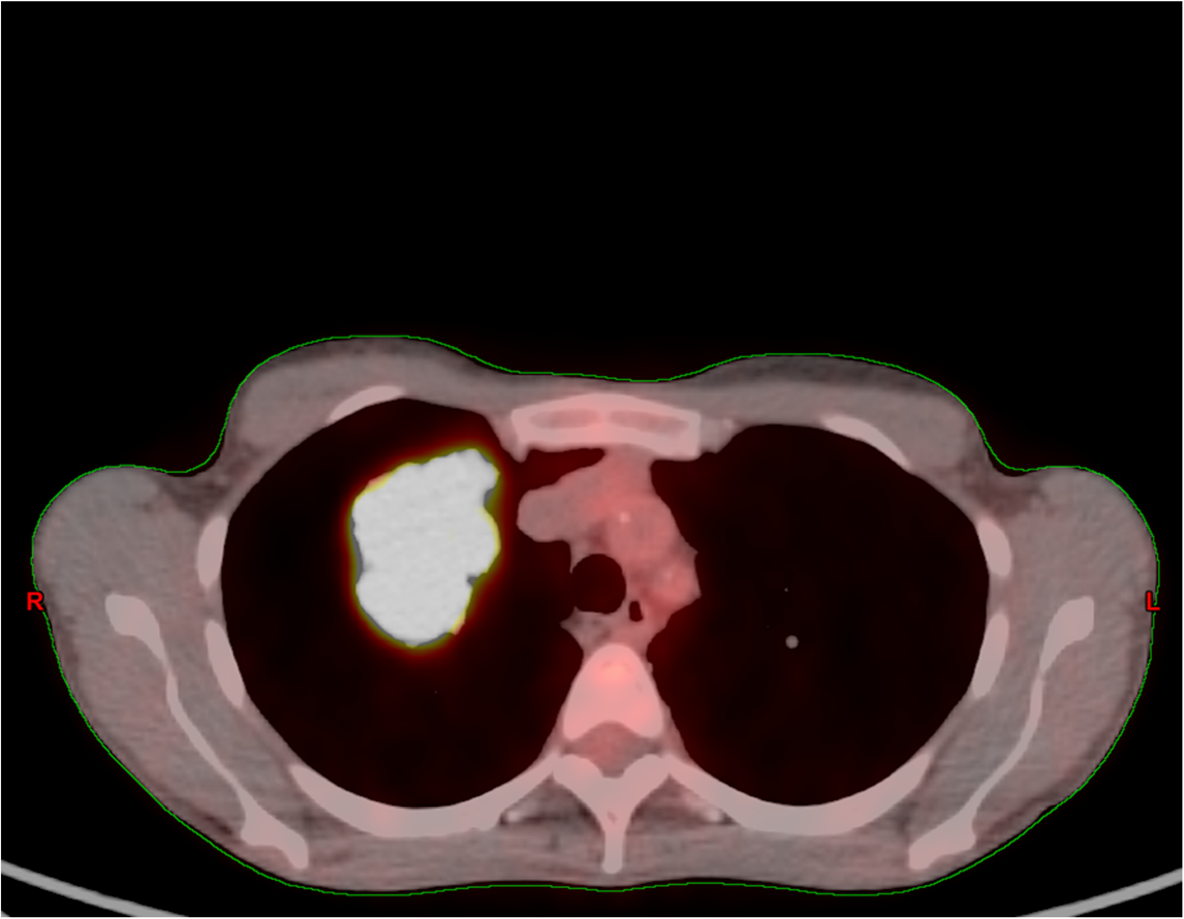
**An axial section of the treatment planning computed tomography scan fused with positron emission tomography.** A right upper lobe lung mass can be seen.

**Figure 2:**
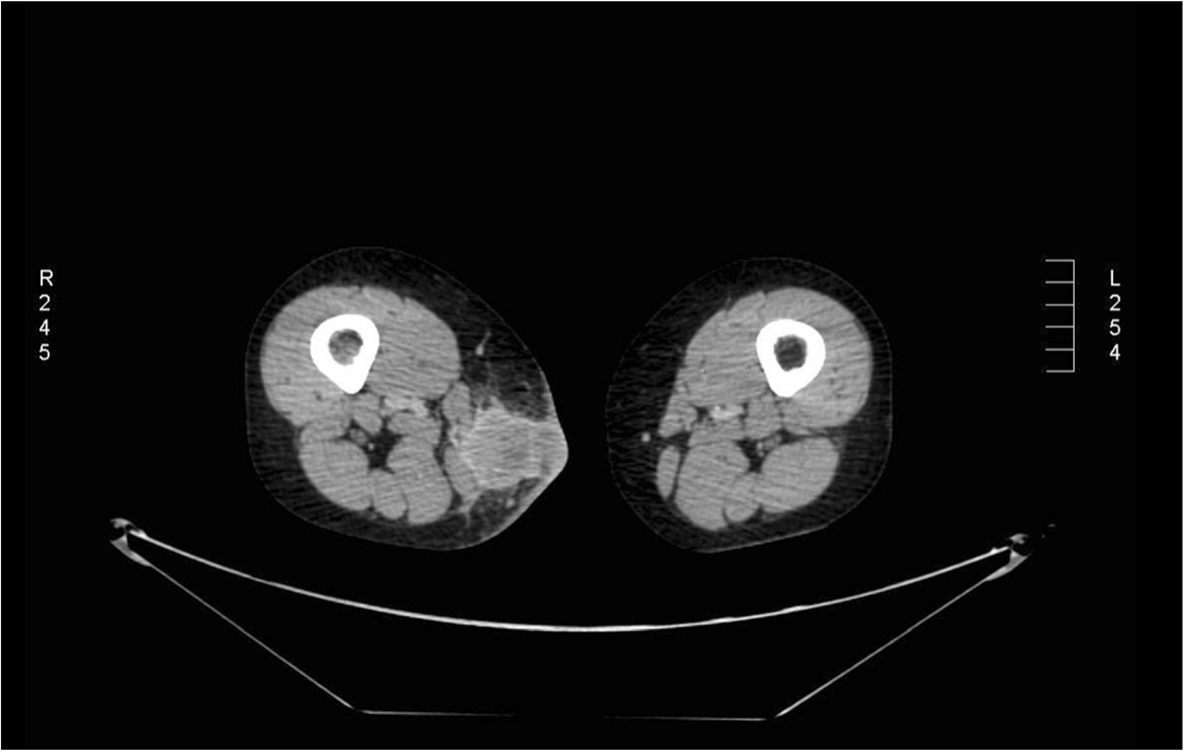
An axial section of a computed tomography scan showing a lesion in the soft tissue of the medial compartment of the right thigh.

**Figure 3:**
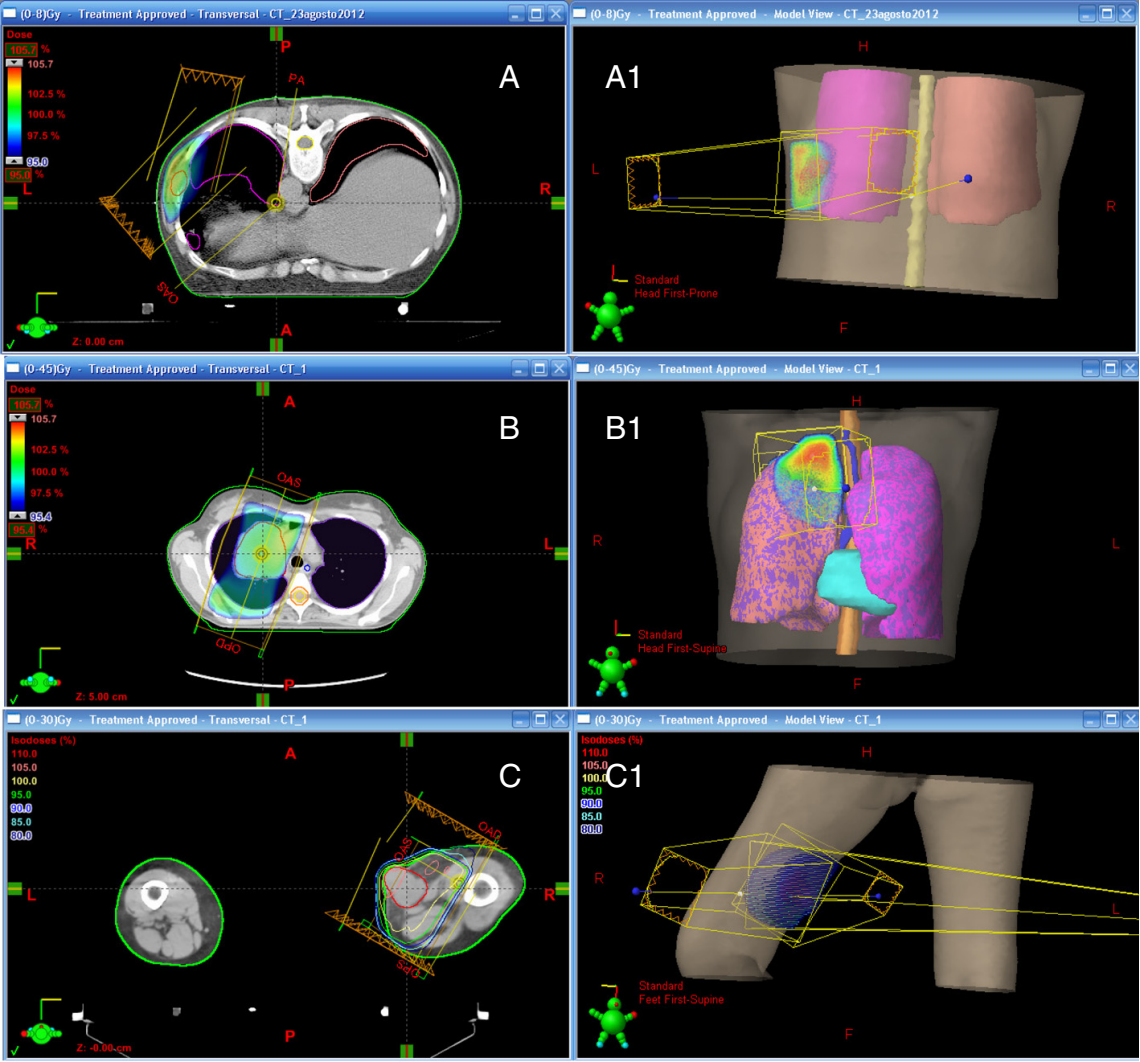
Radiation treatment planning: (A) ninth rib, (B) right upper lobe lung mass, (C) right thigh lesion; (A1), (B1) and (C1) show three-dimensional reconstructions.

A 45-year-old Caucasian woman presented to our facility with a history of right shoulder pain that had persisted for several months and was resistant to medical treatments. She reported no systemic disease. She had been a smoker for 25 years. A contrast-enhanced computed tomography (CT) scan of the chest revealed a right upper lobe lung mass (64×47mm) and mediastinal lymphadenopathy that did not involve the chest wall. CT-guided biopsy of the lung mass provided a histopathological diagnosis of adenocarcinoma (staining for thyroid transcription factor 1 (TTF-1) was positive, staining for p63 was negative). On further staging, brain metastasis was detected (33mm in left parietal region). A whole body 18F-fluorodeoxyglucose (FDG) positron emission tomography (PET) scan was performed and it revealed increased FDG uptake in the primary right upper lobe lung mass, mediastinum and brain (standardized uptake values (SUVs) of 11.5, 6.1 and 13.3, respectively). She underwent neurosurgery and the histological report described the brain lesion as compatible with origin from the primary lung tumor. She received six cycles of systemic chemotherapy consisting of cisplatin and pemetrexed. At one-month follow-up, a PET/CT (Figure 1) scan showed stable FDG uptake in body regions of interest. She received 45Gy (2.5Gy×25 fractions) sequential palliative radiation therapy (RT) on the lung mass, and 30Gy (3Gy×10 fractions) on whole brain, respectively. Figure 1 shows an axial section of the treatment planning CT scan fused with the PET scan. Three months after RT, a CT scan revealed stable lung and brain disease. She was enrolled in an experimental protocol with erlotinib hydrochloride plus ARQ-197 (a selective inhibitor of the c-Met receptor tyrosine kinase)/placebo [4]. One month later, she came to our Radiation Therapy Department for follow-up, and she referred to ha skin lesion first noticed on her right thigh two weeks previously. A physical examination showed phlebitis and edema of the lower limb. For this reason we prescribed low-molecular-weight heparin. Despite multimodality therapy, a new CT total body scan pointed out progressive metastatic disease with a rare lesion of the soft tissue in the medial compartment of the right thigh, infiltrating the skin (Figure 2). Ultrasonography (USG) was performed to evaluate the thigh lesion; it showed an inhomogeneous, hypoechoic image, with irregular margins (50×40mm). This lesion enveloped contiguous superficial vessels, involved the subcutaneous tissue and infiltrated the skin. Fine needle aspiration cytology (FNAC) was performed. After cytological definition as a muscle metastasis consistent with the known primary lung cancer, she was treated with RT to the right thigh mass with 30Gy in 10 fractions. She developed pain in her left hemithorax and a single photon emission computed tomography (SPECT) study showed increased uptake in the area of the ninth rib. A single fraction of RT was delivered (total dose was 8Gy). Figure 3 shows the three treatments. She is alive 18 months on from diagnosis, with metastatic disease; she has no symptoms related to the metastasis and only an ulcerated lesion is visible on her right thigh. The last USG showed a reduction of the lesion (19×7mm). The radiation treatments made reducing the dose of analgesic therapy possible, and resulted in resolution of edema of her leg. RT has improved the quality of life of our patient. Our patient is currently undergoing therapy with gemcitabine.

### Case 2

**Figure 4:**
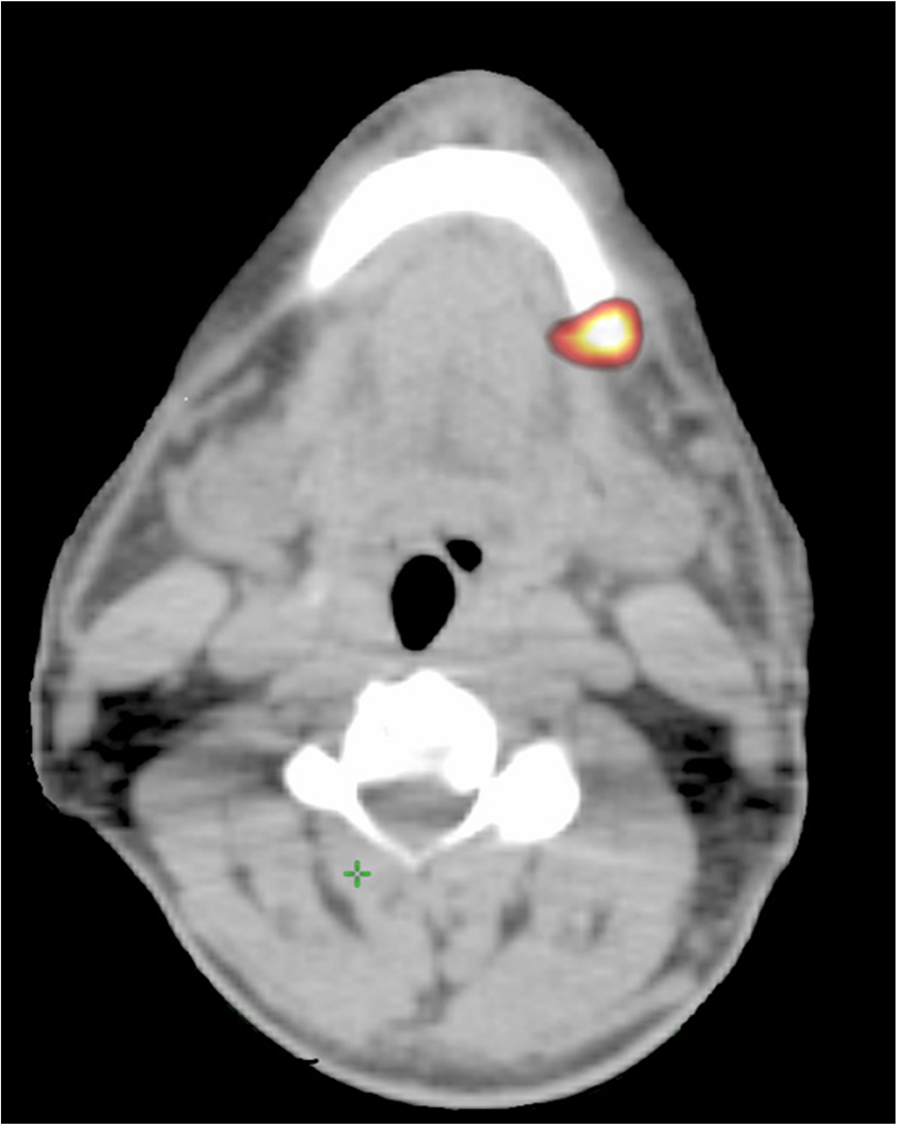
A positron emission tomography/computed tomography scan identified increased fluorodeoxyglucose uptake in the mandible bone.

**Figure 5:**
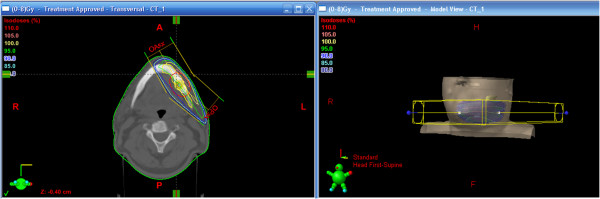
Radiation treatment planning in a single fraction of mandible bone (left); three-dimensional reconstruction (right).

A 61-year-old Caucasian man came to our Radiation Oncology Department with a palpable mass of the mandible on the left side. Our patient reported that he had noted the mandible lesion about two months earlier, with atypical pain. This pain had worsened acutely in the 24 hours before the clinical examination, until it became unbearable. He had been a smoker for more than 30 years. He was apparently in good health otherwise. He reported that he had no systemic disease. A physical examination showed a significant swelling of the left mandible; as our patient had given no history of trauma, the cause of his signs and symptoms was presumed to be related to either infection or malignancy. During the investigation of the pain, a total body CT scan showed a bone lesion of the left mandible (17mm) plus a left lung mass (size at maximum of 37mm), with involvement of the mediastinum. A bone biopsy was performed, showing a metastasis from an epidermoid carcinoma grade 2, consistent with the primary lung cancer. A PET/CT scan identified increased FDG uptake (Figure 4) in the mandible bone (SUV maximum of 6.3) and left lung mass (SUV maximum of 3.2). Our patient’s case was discussed at our multidisciplinary committee: the surgeon’s therapeutic proposal was a double concomitant surgery on the lung mass and on the solitary bone metastasis; the radiotherapist and oncologist recommended radiation therapy on the bone metastasis followed by systemic therapy. Our patient refused the surgical option as worsening his quality of life. He received symptomatic and palliative RT (Figure 5) on the left mandible bone lesion with 8Gy in a single fraction, allowing the initiation of chemotherapy. Three months after the completion of radiation treatment he is alive but he has reported a slight increase in pain on the mandible site, refractory to pharmacological therapy. Due to the reappearance of the pain, we will propose retreatment on the mandible site [5].

## Conclusions

This report presents the cases of two patients affected by NSCLC, metastatic to the right thigh muscle and left mandible. Both patients were treated with RT to the involved area, in addition to systemic chemotherapy. Few data are reported about metastases to muscle from primary lung cancer. Moreover, metastases to soft tissue can be misdiagnosed histologically as primary soft tissue sarcomas. The treatment and prognosis for a metastatic neoplasm of the soft tissue and a primary soft tissue sarcoma are different [6]. Various mechanical factors, such as tissue blood flow, arterial pressure, muscle contraction and trauma, have been described as possible causes for rare metastases to muscle [7]. Often these metastases are diagnosed at different time intervals from the discovery of the primary tumor, being discovered in some cases more than five years later. The most commonly involved sites are the muscles of the trunk, particularly the paraspinal and psoas muscles [8]. In our first patient’s case, however, the region of interest was the lower extremity. Many cases of skeletal muscle metastases described in the literature showed clinical symptoms and signs, such as in our patient with edema and phlebitis. Although several reports underline that these events are not common, a recent study by Haygood *et al*. explores the epidemiology of metastases to skeletal muscle and their detection by PET/CT, concluding that skeletal muscle metastases are not unusual and the most common source is lung cancer [9]. The increasing use of PET/CT has recently led to diagnosis of unsuspected distant/solitary metastases at rare locations, including the colon and extra-ocular muscles [10]. There is no agreement on the optimal therapeutic strategy for muscle metastases from NSCLC, although several therapeutic options can be offered to patients such as radiation therapy, chemotherapy or surgical excision. Unfortunately, the outcome remains poor and the prognosis of these patients with muscle metastasis from NSCLC remains doubtful [11]. In this report, with regard to our first patient, the use of radiation therapy on muscle metastasis can be considered as a potentially successful treatment option, giving patients the possibility to avoid an aggressive treatment such as surgery and reducing the possible side effects.

Likewise, the clinical presentation of mandibular metastases are similar to common conditions such as toothache or, less frequently, temporo-mandibular joint pain, osteomyelitis, or trigeminal neuralgia [12]. Consequently the diagnostic and therapeutic investigation of these patients may be difficult. Metastases to the jaw involve the mandible in 80 percent of cases, and the most common locations for oral metastasis are the molar and pre-molar regions of the mandibular bone. Most studies of oral metastatic disease indicate its predilection for the posterior mandible [12,13] because of its rich blood supply in active areas of hematopoiesis; conversely, in our study, the anterior of the mandible was involved. Metastatic bone lesions are generally determined after the detection of the primary tumor; they may be the first symptom of metastatic disease in approximately 30 percent of cases. Data from the literature reveal that autopsies detected muscle metastases in about 17 percent of patients [14]. In the second case report, we described the clinical history of a patient affected by metastasis to the mandible that disclosed the presence of a silent primary lung cancer. Pruckmayer *et al*. evaluated 763 patients retrospectively who suffered from jaw pain. They concluded that a selected subgroup of individuals, specifically patients with a history of cancer or those not responding to conventional management, should undergo specific investigations such as bone scans to rule out a neoplastic cause [15].

Although radical surgery treatment of the solitary metastatic bone lesion or of muscle metastasis in patients who are oligometastatic and plurimetastatic could be considered as therapeutic options, palliative radiation therapy was offered to our patients obtaining good control of tumor size and pain. Both patients are alive, with follow-up of five and 18 months. Palliative radiation therapy is one of the major contributors to the care of patients with oncological issues, and also, in selected cases, a second radiation therapy treatment is feasible, well tolerated and offers the possibility of symptomatic relief. Our treatments were carefully chosen to maintain the quality of life of our patients in both cases, and we obtained good results without serious side effects although it should be noted the prognosis in metastatic NSCLC remains poor.

## Consent

Written informed consent was obtained from both patients for publication of this case report and any accompanying images. A copy of the written consent is available for review by the Editor-in-Chief of this journal.

## Competing interests

The authors declare that they have no competing interests.

## Authors’ contributions

FMG analyzed the patient data and was a major contributor in writing the manuscript. DA enrolled our patients into the Radiation Therapy Department. GG discussed the cases at our multidisciplinary committee. GDP followed our patients during their treatments. LI elaborated treatment planning and provided writing assistance. MM elaborated treatment planning. FC analyzed the references. AM enrolled our patients into the Medical Oncology Unit, Thoraco-Pulmonary Department. FF performed the histological examination. VR and PM read and approved the final manuscript. All authors read and approved the final manuscript.
